# Surface-Based Analyses of Anatomical Properties of the Visual Cortex in Macular Degeneration

**DOI:** 10.1371/journal.pone.0146684

**Published:** 2016-01-20

**Authors:** Doety Prins, Tina Plank, Heidi A. Baseler, André D. Gouws, Anton Beer, Antony B. Morland, Mark W. Greenlee, Frans W. Cornelissen

**Affiliations:** 1 Laboratory of Experimental Ophthalmology, Department of Ophthalmology, University of Groningen, University Medical Center Groningen, Groningen, the Netherlands; 2 Institute for Experimental Psychology, University of Regensburg, Regensburg, Germany; 3 Department of Psychology, University of York, York, United Kingdom; 4 Centre for Neuroscience, Hull-York Medical School, York, United Kingdom; Chinese Academy of Sciences, CHINA

## Abstract

**Introduction:**

Macular degeneration (MD) can cause a central visual field defect. In a previous study, we found volumetric reductions along the entire visual pathways of MD patients, possibly indicating degeneration of inactive neuronal tissue. This may have important implications. In particular, new therapeutic strategies to restore retinal function rely on intact visual pathways and cortex to reestablish visual function. Here we reanalyze the data of our previous study using surface-based morphometry (SBM) rather than voxel-based morphometry (VBM). This can help determine the robustness of the findings and will lead to a better understanding of the nature of neuroanatomical changes associated with MD.

**Methods:**

The metrics of interest were acquired by performing SBM analysis on T1-weighted MRI data acquired from 113 subjects: patients with juvenile MD (JMD; n = 34), patients with age-related MD (AMD; n = 24) and healthy age-matched controls (HC; n = 55).

**Results:**

Relative to age-matched controls, JMD patients showed a thinner cortex, a smaller cortical surface area and a lower grey matter volume in V1 and V2, while AMD patients showed thinning of the cortex in V2. Neither patient group showed a significant difference in mean curvature of the visual cortex.

**Discussion:**

The thinner cortex, smaller surface area and lower grey matter volume in the visual cortex of JMD patients are consistent with our previous results showing a volumetric reduction in their visual cortex. Finding comparable results using two rather different analysis techniques suggests the presence of marked cortical degeneration in the JMD patients. In the AMD patients, we found a thinner cortex in V2 but not in V1. In contrast to our previous VBM analysis, SBM revealed no volumetric reductions of the visual cortex. This suggests that the cortical changes in AMD patients are relatively subtle, as they apparently can be missed by one of the methods.

## Introduction

Macular degeneration (MD) is a group of retinal diseases which can cause a central visual field defect, due to damage in the macular region. In a previous neuro-imaging study, voxel-based morphometry (VBM) analysis showed volumetric changes in the visual cortex of MD patients with binocular central visual field defects, compared to healthy controls[1]. The goal of the current study was to further characterize these volumetric changes in the same group of MD patients using surface- rather than voxel-based metrics of brain morphology. Surface-based morphometry (SBM) can provide additional information about the brain structure such as cortical thickness, curvature and surface area.[2] Hence, analyses using SBM may provide further insight into the nature of the neuroanatomical changes previously found in MD patients. This is important, as such structural changes might limit future treatments of MD that aim to restore visual function, such as retinal implants, stem-cell treatment and retinal pigment epithelium transplantation.[3,4]

MD can be divided into age-related macular degeneration (AMD) and juvenile macular degeneration (JMD). In AMD, the macula degenerates by the accumulation of drusen in the macular area, which in turn interferes with the retinal metabolism. The degeneration of the macula can cause a central visual field defect.[5–7] Worldwide, AMD is the third most prevalent cause of blindness (8.7%).[8] However, in diverse parts of the world, the prevalence of AMD as a cause of visual impairment or blindness varies. [9–13] JMD is a group of diverse eye diseases, which includes Stargardt’s disease, Best’s vitelliform retinal dystrophy (Best’s disease), cone-rod dystrophy, and central areolar choroidal dystrophy. These diseases start in the early decades of life and are mostly hereditary. The different pathological mechanisms of these diseases all lead to the loss of photoreceptors, and therefore cause a central visual field defect.

If the visual field defect in MD occurs in both eyes and overlaps–which is common–the activity in specific parts along the visual pathways is reduced, potentially causing functional deprivation. This deprivation may be responsible for the structural changes in the visual pathways. Alternatively, changes may be caused by anterograde transsynaptic degeneration, in which damage of the retinal ganglion cells transmits to related neurons, resulting in axonal damage of the visual pathways.

In both AMD and JMD patients, previous studies have found a reduction of the grey matter volume near the occipital pole in the posterior part of the calcarine sulcus.[14,15] This indicates that MD is associated with retinotopic-specific neuronal degeneration of the visual cortex, as the central visual field is projected at the occipital pole. In addition to a decreased grey matter volume, a recent study from our group also showed a decreased white matter volume along the visual pathways in both AMD and JMD patients. Additionally, in AMD, a decreased white matter volume was found outside the visual pathways, particularly in the frontal lobe.[1]

The goal of the current study was to further characterize the previously reported volumetric changes in the visual cortex in MD patients. To do so, we applied SBM to the same group of AMD and JMD patients in which we previously applied VBM and demonstrated changes in grey and white matter volume.[1] Using the surface-based analysis package Freesurfer, we investigated whether these volumetric differences are also reflected in changes in the cortical thickness, mean curvature or cortical surface area of the MD patients compared to age-matched controls. Moreover, we also reassessed grey matter volume using this surface-based approach. Recently, SBM showed a decreased gyrification in albinism in the same areas where VBM had indicated a decreased grey matter volume.[16] Therefore, we hypothesized to find a decreased gyrification in addition to a reduced volume in the visual cortex of MD patients as well.

## Methods

### Ethics statement

This study conformed to the principles of the Declaration of Helsinki, and was approved by the respective medical review board of each centre participating in the study: the Medical Ethical committee of the University Medical Center Groningen in Groningen; the York Neuroimaging Centre Ethics committee and the local National Health Service Ethics committee in York; the Royal Holloway Ethics committee of the University of London and the local National Health Service ethics committee in London; and the Ethical committee of the University of Regensburg in Regensburg. All participants gave written informed consent before participating in the study. Written informed consent from the children enrolled in the study were obtained from both the child and from the respective parent.

### Subjects

For this study, we included 113 subjects, which comprises the same group of subjects that was included in the study of Hernowo *et al*.[1] These subjects were recruited in Groningen (the Netherlands), York and London (United Kingdom) and Regensburg (Germany). Patients were included in this study when they suffered from a binocular visual field defect due to MD. Healthy control subjects had a good visual acuity in both eyes, and were free from visual field defects. All subjects were free from neurological or psychiatric disorders.

Here, we will briefly specify the subjects characteristics. For the more complete description of our subjects, we refer to Hernowo *et al*.[1] We included 34 JMD patients, 24 AMD patients and 55 healthy control subjects. The patients were assigned to either the JMD or the AMD group based on their clinical diagnosis. We split the group of healthy controls into 33 controls for the JMD patients and 22 controls for the AMD patients, based on their age. This makes four subject groups for the analyses: 1) JMD patients, 2) young healthy controls (HCY), age-matched to the JMD patients, 3) AMD patients, and 4) old healthy controls (HCO), age-matched to the AMD patients. JMD patients had a mean age of 40.2 years (range 12–66 years), the HCY subjects had a mean age of 37.4 years (range 13–60 years). AMD patients had a mean age of 75.2 years (52–91 years), the HCO subjects had a mean age of 68 years(61–83 years). Table 1 shows the mean scotoma diameter for the AMD and JMD patients and the mean disease duration for the AMD and JMD patients from Regensburg.

**Table 1:** Patient characteristics.

Characteristics	Values	
	AMD	JMD
Number of subjects	24	34
Scotoma diameter, mean (range), degree	14 (4–25)	20 (3–65)
**Subjects from Regensburg**		
Number of subjects	8	26
Disease duration, mean (range), years	7.6 (1–21)	15.9 (2–42)

Scotoma diameter in degrees of visual angle; mean duration of the disease in the subjects from Regensburg. JMD—juvenile macular degeneration; AMD—age-related macular degeneration.

### Data acquisition

Magnetic resonance images (MRI) were acquired using three different scanners in three centers. However, all acquisitions were of 1 mm x 1 mm x 1 mm resolution. Information on the MRI acquisition has also been described in the previous publication of Hernowo *et al*.[1]

Groningen: the dataset was obtained using an 8-channel phased-array SENSE head coil on a 3.0 Tesla Philips Intera (Eindhoven, The Netherlands) at the Neuroimaging Center, University of Groningen, University Medical Center Groningen. Three-dimensional structural images were acquired using a sequence T1W/3D/TFE-2, 8° flip angle, repetition time (TR) 8.70 ms, echo time (TE) 4.4 ms, matrix size 256 x 256, field of view 230 x 160 x 180, yielding 160 slices.

York: the dataset was obtained using an 8-channel, phased-array head coil on a Siemens Trio 3 Tesla at the Combined Universities Brain Imaging Center, Royal Holloway University of London. Multi-average, whole head T1-weighted anatomical images were acquired using an MDEFT sequence with 16° flip angle, TR 7.90 ms, TE 2.5 ms, matrix size 256 x 256, field of view 176 x 256 x 256, yielding 176 sagittal slices.

Regensburg: the dataset was obtained using a multicoil phased-array head coil on a 3.0 Tesla Allegra Scanner (Siemens, Erlangen, Germany). Whole brain T1-weighted images were obtained using a 3D-MPRAGE sequence, matrix size 256 x 256, field of view of 256 x 256 x 160, yielding 160 slices, using the Alzheimer’s Disease Neuroimaging Initiative (ADNI) sequence (TR = 8.79 ms, TE = 2.6 ms, flip angle 9°).[17]

### Surface-based morphometry

SBM analyses of cortical thickness, mean curvature, surface area and grey matter volume were performed using the Freesurfer image analysis suite (version 5.3.0, available at: http://surfer.nmr.mgh.harvard.edu/). The processing includes removal of non-brain tissue,[18] automated Talairach transformation, intensity normalization,[19] tessellation of the grey/white and grey/cerebrospinal fluid boundaries and automatic correction of topologic inaccuracies,[20,21] surface deformation and inflation,[22,23] registration to a spherical atlas[24] and automatic parcellation of the cortex surface based on gyral and sulcal structures.[25,26] The reconstruction process resulted in of a variety of surface-based data, such as cortical thickness, mean curvature, surface area measurement and grey matter volume.

### Data analysis

We performed both whole brain and region-of-interest (ROI) -based analyses. In the whole brain analyses, we analyzed the cortical thickness and the mean curvature. We studied differences in cortical thickness and mean curvature, and differences in the correlation between age and cortical thickness and the correlation between age and mean curvature between patients and their age-matched controls. To correct for any sources of variance exclusively related to age or scanner location, we added age and scanner location at which the subject was scanned as covariates to this analysis. To correct for multiple comparisons, we applied a false-discovery rate (FDR) value of 0.05.

Cortical thickness was calculated as the shortest distance between the grey/white boundary and the grey/cerebrospinal fluid boundary at each vertex across the cortex in millimeters (mm). Mean curvature was calculated as the mean of the minimum and maximum bending of the surface in each vertex in mm^-1^. Surface area was measured by calculating the surface area size of each triangle, in which the surface was divided by connecting the vertices, in mm^2^. The surface area of a single triangle depends on the number of vertices the cortex was divided into, which in turn depends on the size of the brain. Since it is not yet clear whether this would reflect the actual surface area in whole brain analysis, we decided not to perform whole brain analysis on surface area. Grey matter volume was calculated as the product of the surface area and cortical thickness in mm^3^, therefore we did also not perform a whole brain analyses on grey matter volume.

**Fig 1:**
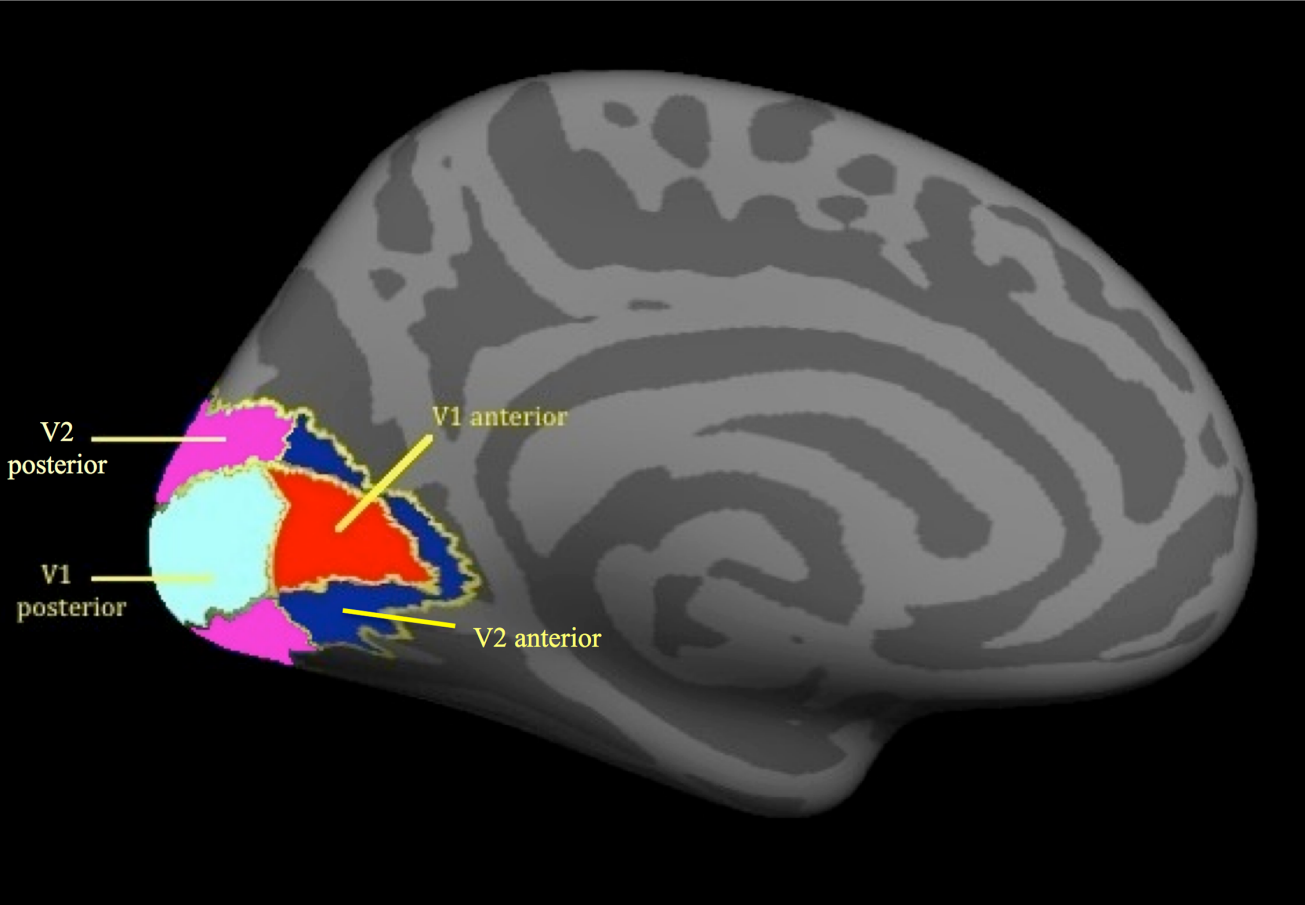
ROIs in the visual cortex. ROIs are depicted on the left hemisphere. Red–V1 anterior; Cyan–V1 posterior; Dark blue–V2 anterior; Magenta–V2 posterior. ROI–region of interest.

For the ROI-analyses we examined the surface area and grey matter volume, as well as the cortical thickness and mean curvature. We defined our ROIs using the Freesurfer labels for the areas V1 and V2. Moreover, we consecutively divided both V1 and V2 in an anterior and a posterior part. We chose to analyze the anterior and posterior parts of V1 and V2 separately because all patients had a central visual field defect. Since the central visual field is retinotopically represented in the occipital pole, we expected the anterior and posterior parts of V1 and V2 not to be equally affected. Therefore, an analysis of the entire V1 and V2 might not present sufficient details on the actual structural changes in our patient groups. Fig 1 depicts the defined ROIs on the left hemisphere of the average brain; the actual analyses were done on the ROIs in both hemispheres combined.

For each ROI, data on cortical thickness, mean curvature, surface area and grey matter volume were extracted using Freesurfer. Differences between patients and controls in the specific ROIs were examined using MANCOVA. The dependent variables were cortical thickness, mean curvature, surface area and grey matter, and the subject groups were entered as a fixed factor. Also in this analysis, age and scanner location were added as covariates.

Specifically in patients from Regensburg, data on disease duration was available. In this subgroup of patients, we analyzed whether there was a correlation between disease duration and cortical thickness, mean curvature, surface area and grey matter for each ROI. We tested this using the Pearson correlation test. Statistical tests were performed in the IBM SPSS Statistics software package, version 20.

## Results

### ROI-based analysis

**Fig 2:**
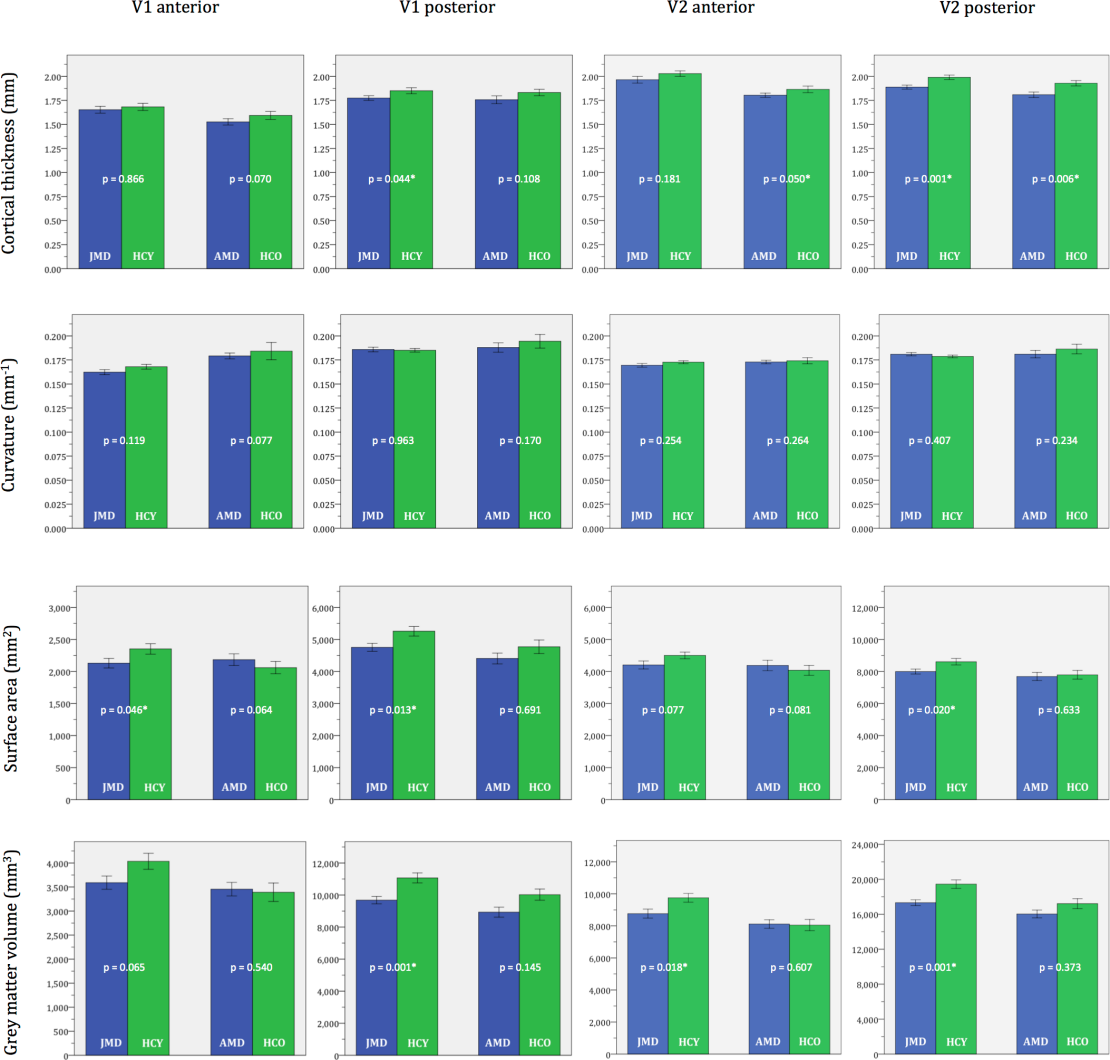
ROI morphometric values. Average cortical thickness, mean curvature, surface area and grey matter volume in the ROIs V1 anterior, V1 posterior, V2 anterior and V2 posterior. The bars show the mean value of the specific metric of the ROI in the particular group. The error bars show ±1 standard errors of the mean. p-values are given. ROI—region of interest; JMD—juvenile macular degeneration; HCY—healthy controls young (age-matched to JMD); AMD—age-related macular degeneration; HCO—healthy controls old (age-matched to AMD). * Significant difference between the patients group and the respective age-matched control group (p < 0.05).

Within the V1 anterior, V1 posterior, V1 anterior and V2 posterior ROIs, we performed analyses of the cortical thickness, mean curvature, surface area and grey matter volume. Fig 2 shows the mean values of the anatomical features for all four groups: the JMD patients, the younger healthy controls, the AMD patients and the older healthy controls. Table 2 presents more details on these values and highlights which features differed significantly between patients and controls in the individual ROIs.

**Table 2:** ROI morphometric values (μ ± σμ).

		JMD	HCY	f-value (df = 1.63)	p-value	AMD	HCO	f-value (df = 1.42)	p-value
**Average cortical thickness (mm)**	V1 anterior	1.653 ± 0.036	1.682 ± 0.037	0.029	0.866	1.527 ± 0.033	1.594 ± 0.042	3.447	0.070
	V1 posterior	1.775 ± 0.024	1.851 ± 0.031	4.227	0.044 *	1.756 ± 0.041	1.832 ± 0.034	2.700	0.108
	V2 anterior	1.965 ± 0.035	2.029 ± 0.029	1.834	0.181	1.804 ± 0.022	1.865 ± 0.035	4.078	0.050 *
	V2 posterior	1.888 ± 0.021	1.991 ± 0.023	11.400	0.001 *	1.809 ± 0.028	1.929 ± 0.028	8.351	0.006 *
**Mean Curvature (mm**^**-1**^**)**	V1 anterior	0.162 ± 0.003	0.168 ± 0.003	2.500	0.119	0.179 ± 0.003	0.184 ± 0.009	3.297	0.077
	V1 posterior	0.186 ± 0.002	0.185 ± 0.002	0.002	0.963	0.188 ± 0.005	0.194 ± 0.007	1.947	0.170
	V2 anterior	0.170 ± 0.002	0.173 ± 0.002	1.323	0.254	0.173 ± 0.002	0.174 ± 0.003	1.282	0.264
	V2 posterior	0.181 ± 0.002	0.179 ± 0.001	0.696	0.407	0.181 ± 0.004	0.186 ± 0.005	1.495	0.234
**Surface area (mm**^**2**^**)**	V1 anterior	2130.29 ± 74.94	2352.67 ± 81.51	4.145	0.046 *	2185.38 ± 90.65	2060.50 ± 95.69	3.607	0.064
	V1 posterior	4753.91 ± 123.91	5256.55 ± 149.70	6.598	0.013 *	4408.08 ± 169.35	4770.41 ± 212.15	0.160	0.691
	V2 anterior	4203.88 ± 124.91	4502.91 ± 104.34	3.225	0.077	4189.17 ± 162.17	4037.82 ± 153.63	3.190	0.081
	V2 posterior	7998.18 ± 151.94	8613.58 ± 204.26	5.718	0.020 *	7688.42 ± 252.08	7795.09 ± 273.75	0.231	0.633
**Grey matter volume (mm**^**3**^**)**	V1 anterior	3590.97± 138.45	4035.21 ± 166.09	3.516	0.065	3455.33 ± 140.77	3391.14 ± 192.45	0.381	0.540
	V1 posterior	9682.56 ± 229.84	11068.33 ± 311.69	11.898	0.001 *	8931.46 ± 312.43	10024.64 ± 347.80	2.205	0.145
	V2 anterior	8764.59 ± 280.49	9750.70 ± 272.10	5.890	0.018 *	8117.08 ± 266.15	8053.18 ± 349.58	0.269	0.607
	V2 posterior	17325.68 ± 331.33	19448.18 ± 485.69	12.028	0.001 *	16031.33 ± 446.11	17215.18 ± 564.76	0.811	0.373

Average cortical thickness, mean curvature, surface area and grey matter volume were extracted from the ROIs V1 anterior, V1 posterior, V2 anterior and V2 posterior. Mean values and standard error of the mean are presented for each parameter and each ROI (f-values, degrees of freedom and p-values are given).

ROI—region of interest; μ - mean; σμ - standard error of the mean; df–degrees of freedom; JMD—juvenile macular degeneration; HCY—healthy controls young (age-matched to JMD); AMD—age-related macular degeneration; HCO—healthy controls old (age-matched to AMD).

* Significant difference between the patients group and the respective age-matched control group (p < 0.05).

In JMD patients, we found a thinner cortex in V1 posterior and V2 posterior, a smaller surface area in V1 anterior, V1 posterior and V2 posterior and a lower grey matter volume in V1 posterior, V2 anterior and V2 posterior compared to age-matched healthy controls (p<0.05). No differences were found in mean curvature measurements.

In AMD patients, we found a thinner cortex in V2 anterior and V2 posterior compared to age-matched healthy controls (p<0.05). We did not find differences in mean curvature, surface area size and grey matter volume.

Furthermore, we tested whether disease duration was correlated with cortical thickness, mean curvature, surface area or grey matter volume in the defined ROIs. Since data on disease duration was only available from patients from Regensburg, we performed these analyses only in these subgroups. In both JMD and AMD patients we found no significant correlation between disease duration and any of the parameters in the ROIs.

### Whole brain analysis

We performed SBM analyses of the cortical thickness and mean curvature in the whole brain in the JMD and in the AMD group, both compared to their age-matched healthy control group. Compared to the healthy controls, we found no significant difference (applying an FDR value of 0.05) neither for the JMD nor for the AMD patient group. Furthermore, we analyzed the correlation between age and cortical thickness and the correlation between age and mean curvature in the whole brain in the JMD and the AMD group, compared to their age-matched control group. Also in this analysis, we found no significant differences (applying an FDR value of 0.05) for neither the JMD nor the AMD patients.

## Discussion

To our knowledge, this is the first study that reports the results of a SBM analysis of the visual cortex of MD patients. The association of MD– a common cause of blindness world-wide–with structural changes in the brain has only recently become clear.[1,14,15,27–32] Previous studies have reported volumetric reductions of grey and white matter along the entire visual pathways of MD patients.[1,14,15] Here, we used a surface-based approach to examine several additional anatomical features of the visual cortex in MD, such as cortical thickness, mean curvature and surface area. This SBM analysis further characterizes the previously reported volumetric changes in the visual cortex of MD patients.

### ROI-based analysis confirms the presence of structural changes in JMD patients

In the JMD patients, compared to age-matched controls, we found a smaller cortical thickness in V1 posterior and V2 posterior, a smaller surface area in V1 anterior, V1 posterior and V2 posterior, and lower grey matter volume in V1 posterior, V2 anterior and V2 posterior. We found no differences in mean curvature.

The majority of the changes were found in the posterior region of the visual cortex, where central vision is represented. This is in agreement with the notion that the central visual field defects may cause–through a loss of activity in the visual pathways–the structural changes. However, the surface area was also reduced in V1 anterior and the grey matter volume was also reduced in V2 anterior. In V2 anterior, the cortical thickness and the surface area were also lower in the JMD patients than in the healthy controls, but these findings did not reach significance (p = 0.181 for cortical thickness; p = 0.077 for surface area). These thinner cortex and smaller surface area, albeit not significant, can together explain the significant changes in grey matter volume in V2 anterior. This suggests that not all structural changes have a direct bearing on retinotopic-specific deprivation. Possibly, spontaneous oscillatory spike bursts of the retinal ganglion cells could partly explain these results. Such spontaneous activity has been reported in retinal degeneration mice and rats.[33–35] Furthermore, MD patients have to rely on their peripheral visual field for their daily tasks. This means that the peripheral visual field has a different function in MD patients than healthy controls. However, previous fMRI studies have shown conflicting results on whether functional changes in the visual cortex appear in MD patients.[27,32,36–39] Nevertheless, even if functional changes do not appear in MD, the changed usage of the peripheral visual field might form an additional explanation for the anatomical changes in the anterior V1 and V2. Together, these findings in the ROI analyses show that our previously reported volumetric reductions of grey matter in the visual cortex of JMD patients are associated with a thinner cortex and a smaller surface area, but not with alterations in the mean cortical curvature.

### Partial confirmation of reduced cortical thickness in AMD patients

In AMD patients, compared to age-matched controls, only cortical thickness in V2 anterior and V2 posterior was reduced. In contrast, in our previous VBM study, in the AMD patients, we found more widespread volumetric reductions of the grey matter in cortical regions including primary visual cortex. The fact that changes in cortical thickness were found in both V2 anterior and V2 posterior suggests that these may not have a direct bearing on a retinotopic-specific deprivation. However, also in AMD the previously suggested possibility of spontaneous activity of the retinal ganglion cells and the different function of the peripheral visual field might influence these anatomical changes.[27,32–39] Additionally, the observed neuroanatomical changes in MD might not only be explained by functional deprivation due to the visual field defect, but also to associated neurodegenerative processes. Specifically, a previously described association between AMD and Alzheimer’s disease might play a role in the neuroanatomical changes.[40–43]

### More widespread structural degeneration in the visual cortex of JMD compared to AMD patients

In the JMD patients we found more widespread cortical thinning, reduction of surface area and volume than in the AMD patients. This could be explained by the fact that–on average–the JMD patients in our study had larger visual field defects and a poorer visual acuity than the AMD patients. As a result, visual deprivation will have been more severe in the JMD patients too. If cortical structural degeneration is related to visual deprivation, AMD patients would be expected to show less extensive reductions than the JMD patients, as indeed we find here. Additionally, due to the ageing of their brains, also the healthy older subjects might show more variability in cortical structure, which can make it more difficult to demonstrate disease-related changes in AMD patients.

### No evidence for a changed cortical gyrification pattern in MD patients

Based on *Bridge et al*., [16] who reported a decreased gyrification in albinism in an area where VBM showed decreased grey matter volume, we expected to find a similar decrease in gyrification also in our group of MD patients in the occipital pole–the area where previously grey matter volumetric reductions were found. However, in our patients, mean curvature is the only measurement that did not show any differences in either of the patient groups, compared to their age-matched healthy controls. This suggests that the cause of cortical degeneration in MD and albinism may be rather different in nature, which may be related to the fact that albinism is a congenital disease, whereas MD develops later in life.

### Whole-brain analysis reveals no structural changes outside of visual cortex

To explore the further presence of anatomical changes, we also performed an exploratory whole brain SBM analysis. We were keen on performing these analyses, because morphological differences might also be present outside of the visual cortex. Specifically, our previous VBM analysis had indicated the presence of white matter volumetric reductions in the frontal lobe in the AMD patients.[1] However, compared to their respective age-matched controls, in neither the JMD nor the AMD patients we found differences in cortical thickness or in mean curvature. As mentioned above, such differences were revealed in the ROI-based SBM analyses in V1 and V2, and they were also more evident in the JMD patients than in the AMD patients. These differences between the results of whole-brain and ROI-analysis can be explained by the fact that the threshold for finding differences in whole-brain analysis was higher than in ROI analysis. The higher threshold in whole brain analysis was due to the correction for multiple comparisons, which is necessary when analysing such amounts of data points. In ROI-analysis it is not necessary to apply correction, since the amount of comparisons is much lower and the specific ROIs were selected based on the likelihood of finding differences based on previous studies

### Comparison of SBM- and VBM-analyses

The surface-based approach that we used here indicates a number of structural changes that are consistent between our present SBM and our previous VBM study, particularly in the JMD patients. However, not all SBM results corroborate the VBM results. Part of the difference may be due to the use of different methodology. SBM applies a different method for segmenting the brain, and also for the calculation of the grey matter volume than VBM. Therefore, the border between grey matter and white matter and the cerebrospinal fluid is defined differently in each method. Consequently, the grey matter volume measurements can also be different. On the one hand, finding changes using such different approaches establishes their robustness. On the other hand, if one of the techniques is more sensitive than the other, subtle cortical changes could simply be missed by one of the methods. SBM might give a more accurate representation of structural differences because it takes the highly folded nature of the cortex into account, which is not the case in VBM analysis. This could also explain some of the discrepancies between the results of our two studies. Likewise, previous studies in a variety of neurological and ophthalmological disorders also applied both types of analyses, and also found some differences in their results in surface-based analyses compared to voxel-wise analysis. SBM does not always find changes in cortical features in areas where VBM found differences in grey matter volume.[16,44–47] The opposite occurs as well: in some studies SBM revealed changes in cortical features in areas where VBM did not uncover volumetric differences.[16,47,48] A limitation of SBM is that at present it is only able to uncover changes in grey matter.

## Limitations

In our present study, the ROI definitions of V1 and V2 were not based on a retinotopic examination in the individual subjects, but on average brain templates, included in Freesurfer. In the individual subjects, this may cause a deviation of the borders of these ROIs from their actual ones, which may have affected our results in the ROI-analysis.

In this study, we combined structural MRI data from three different centers. The different MRI-scanners and setting at each location could potentially have an effect on analyses of the cortical properties. However, there are two reasons that we can exclude such effects. First, we avoid systematic influences of differences in scanner properties by including scanner location as a covariate in all of our analyses. Second, both patients and controls were scanned in all three scanners. An important advantage of combining the data is the large groups of patients and controls that are obtained in this way, which increases study power and allows drawing more robust conclusions.

From the analyses in the JMD patients, we can conclude that in the diseases that we investigated, the presence of a central visual field defect at a young age of onset, is associated with neuro-anatomical changes. However, it is not possible to determine whether such neuro-anatomical changes occur in the separate diseases from the present analyses. We included diseases of different entity in the JMD group for several reasons. First, patients with the different diseases included in the JMD group form small groups separately, from which we would not be able to draw strong conclusions. Second, although the etiology of the separate diseases is different, they have in common the development of a central visual field defect and the age of onset in the first decades of life. Therefore, we are able to draw robust conclusion from these analyses about the association between a central visual field defect which develops in the first decades of life and changes in the brain.

## Future research

A strength of the present study is that we re-examined the same group of subjects with SBM as Hernowo *et al*.[1] did with VBM. Therefore, we were able to compare the outcomes of these two methods, determine the robustness of the structural brain changes in MD, and unravel more details regarding the nature of the previously found volumetric changes. It would be interesting to use both of these methods in the future in a new group of patients, preferably in a longitudinal study. With such a study design it would be possible to track changes in specific anatomical features in individual patients over time, and more precisely determine the relationship between disease progression and severity of the degeneration. In turn, this could guide future research into therapies for MD, which might have to expand their focus from exclusive ocular treatment to combining eye treatment with treatment of neurodegeneration.

With 3T MRI images and the current methods of segmentation of the brain it is possible to examine differences in neuroanatomical properties. However, it is not possible to specify whether changes in the cortex are supragranular, granular or infragranular, which would be particularly interesting for a better understanding of the pathogenesis of the brain changes. In the future, it might be possible to perform such analyses of the cortical layers, with 7T and super-high resolution (9T) MRI scanning. However, it will take some years before these techniques can be applied in large groups of patients, as we did in the present study.

## Summary

In summary, using SBM, we found changes in several anatomical properties in the visual cortex of both JMD and AMD patients, compared to healthy age-matched controls. JMD patients showed a thinner cortex, smaller surface area and lower grey matter volume in V1 and V2. AMD patients showed a thinner cortex in V2 only. These different findings in JMD and AMD patients may be related to the larger visual field defect and poorer visual acuity in JMD patients compared to AMD patients. Adding to our previous VBM study in the same group of subjects, these results confirm the cortical degeneration in MD patients and indicate that the chance for successful therapeutic restoration of functional vision reduces with disease progression.
